# Risk of Regional Recurrence After Negative Repeat Sentinel Lymph Node Biopsy in Patients with Ipsilateral Breast Tumor Recurrence

**DOI:** 10.1245/s10434-018-6384-y

**Published:** 2018-03-01

**Authors:** Ingrid G. M. Poodt, Guusje Vugts, Adriana J. G. Maaskant-Braat, Robert-Jan Schipper, Adri C. Voogd, Grard A. P. Nieuwenhuijzen, Sentinel Node and Recurrent Breast Cancer (SNARB) study group, R. M. H. Roumen, E. J. T. Luiten, E. J. T. Rutgers, M. T. F. D. Vrancken-Peeters, M. Bessems, J. M. Klaase, S. Muller, A. B. Francken, T. Van Dalen, L. Jansen, S. A. Koopal, Y. L. J. Vissers, M. L. Smidt, J. W. S. Merkus, C. M. E. Contant, P. H. Veldman, E. M. H. Linthorst-Niers, J. R. van der Sijp, O. R. Guicherit, L. B. Koppert, A. M. Bosch, L. J. A. Strobbe, M. S. Schlooz-Vries, I. E. Arntz, J. A. van Essen, J. W. D. de Waard, B. C. Vrouenraets, B. van Ooijen

**Affiliations:** 1 Department of Surgery, Catharina Hospital Eindhoven, Eindhoven, The Netherlands; 2 Department of Surgery, Máxima Medical Center, Eindhoven, The Netherlands; 3 Department of Epidemiology, Maastricht University Medical Center, Maastricht, The Netherlands; 4 Department of Research, Netherlands Comprehensive Cancer Organisation (IKNL), Utrecht, The Netherlands; 5 GROW-School for Oncology and Developmental Biology, Maastricht University Medical Center, Maastricht, The Netherlands

## Abstract

**Background:**

Repeat sentinel lymph node biopsy (rSLNB) has increasingly been used in patients with ipsilateral breast tumor recurrence (IBTR). The safety in terms of regional disease control after this procedure remains unclear. This study evaluates occurrence of regional recurrence as first event in patients with IBTR and negative rSLNB, treated without additional lymph node dissection.

**Patients and Methods:**

Data were obtained from the Sentinel Node and Recurrent Breast Cancer (SNARB) study. In 201 patients, tumor-negative rSLNB was obtained without performing additional lymph node dissections.

**Results:**

With median follow-up of 4.7 (range 0.9–12.7) years, regional recurrence occurred after median time of 3.0 (range 0.4–6.7) years in 4.5% (*N* = 9) of patients as first event after IBTR and rSLNB. In four of these nine patients, the site of recurrence was in concordance with the anatomical location of rSLNB. Two of the nine recurrences were reported in the ipsilateral axilla, resulting in an ipsilateral axillary regional recurrence rate of 1.0%. In the other seven patients, regional recurrence occurred in aberrant basins. Univariable analysis showed that triple-negative IBTR and lower amount of radioactive-labeled tracer (^99m^technetium) used during rSLNB were associated with developing regional recurrence as first event after negative rSLNB (*P* < 0.05).

**Conclusions:**

The risk of developing regional recurrence after negative rSLNB is low. The low relapse rate supports the safety of rSLNB as primary nodal staging tool in IBTR. The time has come for clinical guidelines to adopt rSLNB as axillary staging tool in patients with IBTR.

**Electronic supplementary material:**

The online version of this article (10.1245/s10434-018-6384-y) contains supplementary material, which is available to authorized users.

During recent decades, sentinel lymph node biopsy (SLNB) has emerged and is currently accepted as the new standard practice for axillary staging in patients with primary breast cancer.^1^ Following the growing confidence in the efficacy of SLNB, questions were raised regarding use of this procedure in patients with ipsilateral breast tumor recurrence (IBTR). To date, there is no standard practice regarding axillary staging in IBTR,^2^ hence several studies have evaluated repeat SLNB (rSLNB) in the recurrent setting. These studies showed feasibility of repeat sentinel node in approximately 65% of cases and revealed a role for this procedure in tailoring adjuvant treatment plans.^3^,^4^ However, safety in terms of regional disease control and regional lymph node recurrence after rSLNB in patients with IBTR remains unclear.

In primary breast cancer, regional disease control after SLNB without completion axillary lymph node dissection (cALND) has been investigated critically. False-negative rates of approximately 5%, very low regional recurrence rates, and survival rates comparable to those following ALND justified the conclusion that ALND could be safely omitted in sentinel lymph node-negative patients,^1^,^5^^–^7 thereby sparing patients with primary breast cancer from the morbidity associated with ALND.^8^,^9^

For patients with IBTR and tumor-negative rSLNB, clinically relevant treatment changes were also observed. With negative predictive value of 94%, omission of additional lymph node dissection was assumed to be safe.^3^ Nonetheless, long-term follow-up data on regional recurrence after negative rSLNB have not yet been published. For rSLNB to become an equivalent standard of care in the IBTR setting as well, it is imperative to ensure high regional disease control. Therefore, the aim of this study is to evaluate occurrence of regional recurrence as first event after negative rSNLB in patients with nonmetastatic IBTR, treated with curative intent and without cALND.

## Patients and Methods

### SNARB Study Design

The SNARB study is a multicenter national registration study in which 36 Dutch hospitals participated. In the period from February 2008 to July 2011, data of 150 patients with recurrent breast cancer were prospectively entered into the database.^10^ Subsequently, from August 2011 to December 2014, data from 386 additional patients were retrospectively entered into the database. The additional data were derived from 29 of the 36 initial participating hospitals. Patients with clinically apparent ipsilateral or contralateral lymph node metastases and patients with distant metastases were excluded.^3^ A total of 536 patients, over 18 years old with operable locally recurrent breast cancer and staged with rSLNB, were included. During rSLNB, the dual-mapping technique with both ^99m^techneticum and blue dye was used. From January 2017 to July 2017, follow-up data of the 536 included SNARB patients were collected and entered into the database.

### Patients

Patients with IBTR and successful rSLNB were considered eligible for inclusion. Patients who underwent additional axillary lymph node dissection as validation procedure after detection of a negative sentinel lymph node were excluded. Patients with either micro- or macrometastases in their sentinel lymph node were excluded. Sentinel lymph nodes containing isolated tumor cells (ITC) (small clusters of cells < 0.2 mm and/or fewer than 200 cells) were classified as node negative.

### Regional Recurrence

The primary endpoint of this study was regional lymph node recurrence as first event after curative treatment of IBTR. Regional recurrence was defined as any evidence of disease found in ipsilateral intramammary nodes, ipsi- and contralateral internal mammary nodes, ipsi- and contralateral axillary nodes, and ipsi- and contralateral infra- and supraclavicular nodes. Lymph node recurrences found outside these nodal basins were defined as distant metastatic disease. In the recently published Maastricht Delphi Consensus statement on the definition of regional events, only ipsilateral nodal recurrences (either axillary or in other ipsilateral nodal basins) were considered as regional recurrences.^11^ Based on earlier rSLNB studies,^3^,^12^ we state that the definition of a regional recurrence after IBTR should be broadened and should also include contralateral nodes, since lymphatic drainage towards these basins is common.^4^,^13^,^14^ Therefore, we considered contralateral events as regional recurrences.

Regional recurrences were registered if they occurred as first event after negative rSLNB or when diagnosed concurrently with local recurrences in the previous treated breast. Patients with regional recurrence coincident with or after diagnosis of distant disease were not reported as having regional recurrence as first event. In patients with second IBTR or newly diagnosed contralateral breast tumor as first event without clinically (i.e., physical examination or after imaging studies) relevant regional lymph node metastases, possible lymph node metastases found during a second rSLNB were not regarded as regional recurrence.

### Follow-Up

General practitioners were actively contacted for additional follow-up information when hospital records showed no outpatient clinic visits for more than 1 year. Date of last follow-up was documented as last visit to the outpatient clinic, date of visit to the general practitioner, or date of death in case the patient had deceased.

Follow-up time was defined as time from date of surgery for initial IBTR to date of last follow-up. Time to regional recurrence was defined as time between treatment of IBTR and date of diagnosis of regional recurrence as first event after IBTR.

### Statistics

The variables used (see Supporting Information) were compared between patients with regional recurrence as first event and patients with no regional recurrence. Statistical significance was tested using Pearson Chi square test and Fisher exact test for categorical variables. For continuous variables, Mann–Whitney *U* test or independent sample *t* test was used when appropriate. Two-sided *P*-value < 0.05 was considered statistically significant. Survival analysis, using the Kaplan–Meier method, was performed to calculate the 5-year risk of regional recurrence after IBTR. Data analysis was performed using SPSS version 24 (SPSS Inc., Chicago, IL, USA).

## Results

### Patients

Follow-up data were collected for 536 patients. As 21 patients were lost to follow-up due to file loss, emigration, or loss of informed consent, 515 patients remained available for analysis. Of these 515 patients, 230 patients had successful negative rSLNB, of whom 29 patients were excluded since they underwent additional lymph node dissection. The median age of the remaining 201 patients at time of IBTR was 63.5 (range 34–87) years. The median time from primary surgery to diagnosis of IBTR was 8.5 (range 0.4–30) years. After treatment of IBTR, 22.4% of patients underwent (re)irradiation to the chest wall or regional lymph node basins (*N* = 45). Of all patients, 63.2% received adjuvant systemic treatment (*N* = 127). Adjuvant endocrine therapy was administered in 56.7% (*N* = 114) and adjuvant chemotherapy in 16.9% of the patients (*N* = 34). Patient characteristics are summarized in Table 1.

**Table 1:** Clinicopathological characteristics of all patients with IBTR and negative repeat sentinel lymph node biopsy, without additional lymph node dissection (*N* = 201)

	Total group (*N* = 201)	Regional recurrence (*N* = 9)	No regional recurrence (*N* = 192)	*P*-value
Age primary tumor, median years (range)	51.0 (26–80)	49.0 (40–69)	51.0 (26–80)	0.758
Age primary tumor, years				0.719
< 35	8 (4.0%)	–	8 (4.2%)	
35–59	137 (68.5%)	6 (66.7%)	131 (68.6%)	
60–69	45 (22.5%)	3 (33.3%)	42 (22.0%)	
≥ 70	10 (5.0%)	–	10 (5.2%)	
Primary surgery				0.679
Mastectomy	38 (18.9%)	2 (22.2%)	36 (18.8%)	
Breast-conserving surgery	163 (81.1%)	7 (77.8%)	156 (81.3%)	
Primary SN				0.411
Negative	88 (43.8%)	2 (22.2%)	86 (44.8%)	
Positive	16 (8.0%)	1 (11.1%)	15 (7.8%)	
No SN	97 (48.3%)	6 (66.7%)	91 (47.4%)	
Primary axillary surgery				0.512
No axillary staging	18 (9.0%)	2 (22.2%)	16 (8.3%)	
SN negative	86 (42.8%)	2 (22.2%)	84 (43.8%)	
SN positive, cALND	13 (6.4%)	1 (11.1%)	12 (6.3%)	
SN positive, no cALND	3 (1.5%)	–	3 (1.6%)	
ALND	81 (40.1%)	4 (44.4%)	77 (40.1%)	
Primary nodal status				0.645
Negative	145 (72.1%)	6 (66.7%)	139 (72.4%)	
Positive	31 (15.4%)	1 (11.1%)	30 (15.6%)	
Unknown	25 (12.4%)	2 (22.2%)	23 (12.0%)	
Primary tumor size				0.110
< 20 mm	116 (57.7%)	5 (55.6%)	111 (57.8%)	
21–50 mm	33 (16.4%)	1 (11.1%)	32 (16.7%)	
> 50 mm	3 (1.5%)	1 (11.1%)	2 (1.0%)	
Unknown	49 (24.4%)	2 (22.2%)	47 (24.5%)	
Primary tumor grade				0.071
I	38 (18.9%)	–	38 (19.8%)	
II	47 (23.4%)	–	47 (24.5%)	
III	30 (14.9%)	2 (22.2%)	28 (14.6%)	
Unknown	86 (42.8%)	7 (77.8%)	79 (41.1%)	
Receptor status of primary tumor				0.017
Triple negative	8 (4.0%)	1 (11.1%)	7 (3.6%)	
HR− Her2+	2 (1.0%)	1 (11.1%	1 (0.5%)	
HR+ Her2+	3 (1.5%)	–	3 (1.6%)	
HR+ Her2−	58 (28.9%)	1 (11.1%)	57 (29.7%)	
Unknown	130 (64.7%)	6 (66.7%)	124 (64.6%)	
Hormone status primary tumor				0.270
ER and PR negative	18 (9.0%)	2 (22.2%)	16 (8.3%)	
ER/PR positive	106 (52.7%)	3 (33.3%)	103 (53.6%)	
Unknown	77 (38.3%)	4 (44.4%)	73 (38.0%)	
Time from primary surgery to IBTR diagnosis				
Median, months (range)	106.5 (4–361)	143.0 (15–213)	105.0 (4–361)	0.902
Median, years (range)	8.5 (0–30)	11.0 (1–17)	8.0 (0–30)	0.940
< 2 years	19 (9.5%)	2 (22.2%)	17 (8.9%)	0.414
2.1–5 years	39 (19.4%)	1 (11.1%)	38 (19.8%)	
5.1–10 years	50 (24.9%)	1 (11.1%)	49 (25.5%)	
> 10 years	92 (45.8%)	5 (55.6%)	87 (45.3%)	
Age IBTR, median years (range)	63.5 (34–87)	65.0 (41–76)	63.0 (34–87)	0.658
Age IBTR, years				0.951
< 35	1 (0.5%)	–	1 (0.5%)	
35–59	72 (35.8%)	4 (44.4%)	68 (35.4%)	
60–69	74 (36.8%)	3 (33.3%)	71 (37.0%)	
≥ 70	54 (26.9%)	2 (22.2%)	52 (27.1%)	
Location IBTR				0.679
Breast	163 (81.1%)	7 (77.8%)	156 (81.3%)	
Mastectomy scar or chest wall	38 (18.9%)	2 (22.2%)	36 (18.8%)	
Repeat SN aberrant				0.873
Yes	97 (48.3%)	5 (55.6%)	92 (47.9%)	
No	102 (50.7%)	4 (44.4%)	98 (51.0%)	
Unknown	2 (1.0%)	–	2 (1.0%)	
Repeat SN tracer amount, MBq median (range)	109 (20.0–385.0)	80.0 (30.0–117.0)	110.0 (20.0–385.0)	0.044
Tumor size IBTR				0.663
< 20 mm	145 (72.1%)	8 (88.9%)	137 (71.4%)	
21–50 mm	34 (16.9%)	1 (11.1%)	33 (17.2%)	
> 50 mm	2 (1.0%)	–	2 (1.0%)	
Unknown	20 (10.0%)	–	20 (10.4%)	
Tumor grade IBTR				0.035
I	40 (19.9%)	–	40 (20.8%)	
II	85 (42.3%)	2 (22.2%)	83 (43.2%)	
III	64 (31.8%)	5 (55.6%)	59 (30.7%)	
Unknown	12 (6.0%)	2 (22.2%)	10 (5.2%)	
Receptor status IBTR				0.002
Triple negative	25 (12.4%)	5 (55.6%)	20 (10.4%)	
HR− Her2+	6 (3.0%)	–	6 (3.1%)	
HR+ Her2+	11 (5.5%)	–	11 (5.7%)	
HR+ Her2−	129 (64.2%)	4 (44.4%)	125 (65.1%)	
Unknown	30 (14.9%)	–	30 (15.6%)	
Radiotherapy IBTR				1.000
Yes	45 (22.4%)	2 (22.2%)	43 (22.4%)	
No	156 (77.6%)	7 (77.8%)	149 (77.6%)	
Systemic therapy IBTR				0.728
Yes	127 (63.2%)	5 (55.6%)	122 (63.5%)	
No	74 (36.8%)	4 (44.4%)	70 (36.5%)	
Endocrine therapy IBTR				
Yes	114 (56.7%)	5 (55.6%)	110 (57.3%)	0.505
No	87 (43.3%)	4 (44.4%)	82 (42.7%)	
Chemotherapy IBTR				1.000
Yes	34 (16.9%)	1 (11.1%)	33 (17.2%)	
No	167 (83.1%)	8 (88.9%)	159 (82.8%)	

Univariable analyses compared patients with regional recurrence (*N* = 9) and patients without regional recurrence (*N* = 192)

*IBTR* ipsilateral breast tumor recurrence, *ALND* axillary lymph node dissection, *cALND* completion axillary lymph node dissection, *SN* sentinel node, *mm* millimeter, *HR* hormone receptor, *ER* estrogen*, PR* progesterone, *MBq* megabecquerel*, Her2* human epidermal growth receptor 2

### Regional Recurrence

With median follow-up of 4.7 (range 0.9–12.7) years from IBTR, nine patients were diagnosed with regional recurrence as first event after negative rSLNB (4.5%). These nine regional recurrences occurred after median time of 3.0 (range 0.4–6.7) years. Therefore, the overall regional recurrence rate as first event was 4.5%, with 5-year regional recurrence-free rate of 95.4% [95% confidence interval (CI) 91.9–98.9%]. Of the nine patients with regional recurrence, two patients experienced regional recurrence in the ipsilateral axilla, resulting in an ipsilateral axillary recurrence rate of 1% (Table 2).

**Table 2:** Regional recurrence after negative repeat sentinel lymph node biopsy, without additional lymph node dissection

Regional recurrence	Follow-up IBTR (months)	rSN location LM	rSN location surgical harvested	DFI 1st to 2nd tumor (months)	Primary axillary staging	Primary adjuvant (RT, CT, HT)	Secondary adjuvant (CT, RT, HT)
Ipsilateral axilla	59	Ipsilateral axilla	Ipsilateral axilla	61	None	RT breast	HT
Ipsilateral axilla	43	Contralateral axilla	Contralateral axilla	19	SN+, cALND	RT breast, RT axilla n.a., CT	None
Ipsilateral Supraclavicular	40	Ipsilateral internal mammary chain/intramammary	Ipsilateral axilla	143	ALND	None	RT chest, HT
Ipsilateral supraclavicular	65	Ipsilateral internal mammary chain/intramammary	Ipsilateral internal mammary chain	187	ALND	RT breast	HT
Ipsilateral supraclavicular	9	Ipsilateral axilla	Ipsilateral axilla	15	SN−	None	RT chest
Ipsilateral internal mammary chain	4	Ipsilateral internal mammary chain/intramammary	Ipsilateral internal mammary chain	196	None	RT breast	None
Contralateral axilla	80	Contralateral axilla	Contralateral axilla	213	ALND	RT breast, RT axilla n.a.	CT
Contralateral axilla	15	Ipsilateral axilla	Ipsilateral axilla	30	SN–	RT breast, HT	HT
Contralateral axilla	9	Contralateral axilla	Contralateral axilla	180	ALND	RT breast	None

*DFI* disease-free interval, *IBTR* ipsilateral breast tumor recurrence, *rSN* repeat sentinel node, *LM* lymphoscintigram, *RT* radiotherapy, *CT* chemotherapy, *HT* hormone therapy, *SN*+ sentinel node positive, *cALND* completion axillary lymph node dissection, *ALND* axillary lymph node dissection, *SN*− sentinel node negative, *n.a*. not available

Within the nine patients with regional recurrence, six recurrences were symptomatic (i.e., patients visited the outpatient clinic with lymph node swelling or other localized complaints in an interval between planned follow-up points). The other three recurrences were detected during routine follow-up: two patients during scheduled echography and one patient during scheduled positron emission tomography scan (Table 2).

### Location of Regional Recurrence

Two patients experienced ipsilateral axillary recurrence. One of these patients underwent breast surgery alone (without SLNB or ALND) at the time of the primary breast tumor. During treatment for IBTR, the negative rSLNB was located in the ipsilateral axilla and completion ALND was omitted. Thus, this patient had a relatively intact ipsilateral axilla and developed an ipsilateral axillary recurrence 59 months after treatment for IBTR. The other patient received ALND during primary treatment. At the time of IBTR, the negative rSLNB was located in the contralateral axilla. Forty-three months after IBTR, an ipsilateral axillary recurrence, localized near the subscapular muscle, was diagnosed.

The remaining seven patients developed nodal recurrence outside of the ipsilateral axilla: three in the ipsilateral supraclavicular basin, one in the ipsilateral internal mammary chain, and three in the contralateral axilla. In four of the nine patients (44.4%), the site of regional recurrence was in concordance with the site of the rSLNB on lymphoscintigraphy and during rSLNB surgery (one ipsilateral axilla, one ipsilateral internal mammary chain, and two contralateral axilla).

### Adjuvant Radiotherapy to the Regional Lymph Node Basins

Of all patients, 88.1% (*N* = 177) were primarily treated with adjuvant radiotherapy (for detailed information on location of radiotherapy, see Supporting Information). After treatment of IBTR, 22.4% (*N* = 45) of patients underwent (re)irradiation, of whom 35 patients received radiotherapy to the chest wall, seven patients to the breast, and one to the chest wall and infraclavicular region because of an aberrant node on lymphoscintigram, while in two patients the region was unknown. Two of the nine patients diagnosed with regional recurrence after IBTR received radiotherapy to the chest wall (Table 2).

### Comparison of Variables Between Patients With and Without Regional Recurrence as First Event After IBTR

Comparing patients who developed regional recurrence with those who did not, we did not find significant differences between the groups regarding disease-free interval (DFI), age or tumor size during primary and recurrent breast cancer (Table 1). Likewise, there were no significant differences in administration of adjuvant therapy following IBTR between the two patient cohorts.

Of the nine patients with regional recurrence as first event after negative rSLNB, 55.4% had a triple-negative recurrent tumor compared with 10.4% of the patients without regional recurrence as first event (*P* = 0.002). Furthermore, grade III IBTR was found in 55.6% of patients with regional recurrence compared with 30.7% of patients without regional recurrence (*P* = 0.035). Lastly, patients with regional recurrence as first event were injected with a significantly lower amount of radioactively labeled tracer (^99m^technetium) during rSLNB (median 80.0 MBq) compared with patients without regional recurrence as first event (median 110.0 MBq) (*P* = 0.044) (Table 1).

## Discussion

Data from this study showed that the risk of developing regional recurrence after negative rSLNB in patients with IBTR is low. The low relapse rate supports the safety of rSLNB as nodal staging procedure in the IBTR setting.

After median follow-up of 4.7 (range 0.9–12.7) years, regional recurrence occurred in 4.5% of patients after negative rSLNB. To date, other studies reporting on regional recurrence after negative rSLNB, other than ipsilateral axillary recurrences only, are limited^2^,^12^,^15^^–^27 (Table 3). As shown in Table 3, the number of patients in all these studies were relatively small; moreover, definitions of regional recurrence could not be deduced from the articles. Therefore, comparison of these results with our regional recurrence rate of 4.5% could not be made.

**Table 3:** Articles describing regional recurrence in patients with IBTR and negative repeat sentinel lymph node biopsy, without additional lymph node dissection

Author	Patients (*N*)	Follow-up after IBTR (months)	Regional recurrence	Ipsilateral axillary recurrence
Agarwal et al.^15^	1	25	0 (0%)	0 (0%)
Boughey et al.^17^	8	Median 13 (of 13 patients)	–	0 (0%)
Roumen et al.^24^	2	Mean 14	0 (0%)	0 (0%)
Barone et al.^16^	14	Mean 15 (of 19 patients)	–	0(0%)
Port et al.^23^	31	Mean 26.4 (of 115 patients)	–	0 (0%)
Cox et al.^18^	36	Mean 26 (of 56 patients)	–	0(0%)
Karam et al.^20^	7	Mean 33.3 (of 11 patients)	–	1/7 (14.3%)
Kaur et al.^22^	3	Mean 21.6 (of 45 patients)	–	0 (0%)
Derkx et al.^2^	2	Mean 12	0 (0%)	0 (0%)
Tokmak et al.^25^	5	Mean 27 (of 6 patients)	0 (0%)	0 (0%)
Matsumoto et al.^27^	26	Median 40.3 (of 28 patients)	–	0 (0%)
Intra et al.^12^	171	All 60 (of 196 patients)	–	8/212 (3.9%)^a^
Uth et al.^26^	47	Median 38 (of 144 patients)	–	0 (0%)
Karanlik et al.^21^	15	Mean 36 (of 39 patients)	0 (0%)	0 (0%)
Johnson et al.^19^	8	Median 55.5 (of 12 patients)	1/8 (12.5%)^b^	0 (0%)
15 Articles	376 patients	Range 12–60 (1–3 years)	1/33 (3.0%)	9/376 (2.4%)

*DFI* disease-free interval, *IBTR* ipsilateral breast tumor recurrence

^a^Intra et al. including 25 positive rSLNB with cALND

^b^Johnson et al.: internal mammary node recurrence

In the primary setting, the highest incidence of axillary recurrence occurred between 24 and 42 months.^28^ Recently, Geurts et al. published data about first and second recurrences after curative treatment of primary breast cancer, reporting that the maximum risk of regional recurrence after IBTR was reached within the first year following treatment.^29^ In this study with median follow-up of 4.7 years, sufficient time has elapsed to assume that, after a relatively long follow-up period, the risk of developing regional recurrence after negative rSLNB is low.

In the recent past, performance of ipsilateral ALND in the setting of IBTR was considered as standard care for optimal regional disease control. Therefore, the very low rate of ipsilateral axillary recurrence (1.0%) after negative rSLNB in this study is most interesting. As shown in Table 3,^2^,^12^,^15^^–^27 other studies reporting on ipsilateral axillary recurrence after negative rSLNB showed results that vary between 0 and 9%. Intra et al. published a study on rSLNB with a relatively large number of patients. In that study, an ipsilateral axillary recurrence rate of 3.9% was described, during median follow-up of 5 years.^12^ However, that rate was observed in a cohort of patients with negative repeat sentinel lymph nodes and positive repeat sentinel lymph nodes followed by performance of cALND.

During introduction of the SLNB procedure in the primary setting, an ipsilateral axillary recurrence rate of 5% was accepted to replace cALND by SLNB as standard axillary staging tool.^30^ Later on, Wely et al. reported an even lower ipsilateral axillary recurrence rate of 1.6% after median follow-up of 77 months.^28^ Analogous to these percentages, it seems acceptable that the 1.0% ipsilateral axillary recurrence rate reported herein justifies replacement of ipsilateral ALND by rSLNB in case of clinically node-negative IBTR.

After radiotherapy and surgery of the breast and/or axilla, drainage in rSLNB outside the ipsilateral axilla is described in 18–70% of patients.^3^,^23^,^31^ In this cohort of patients, 48.3% of the rSLNBs were located in an aberrant lymph node station. With the visualization of aberrant lymph drainage in IBTR patients, it is assumed that rSLNB is a more accurate staging method than ipsilateral ALND.^3^ Hence, for at least 48% of our patients, ipsilateral ALND would not have been an accurate staging tool and the aberrant rSNs would have remained unnoticed in absence of rSLNB. With 56% of patients with regional recurrence having an aberrant rSN, it could be hypothesized that one should (re)irradiate aberrant basins on a preventive basis, despite the node-negative outcome. However, this seems to be overtreatment, since the aberrant regional recurrence rate is very low.

Only the 1% of patients with ipsilateral axillary recurrence after rSLNB could have had a possible benefit from ALND. The other seven (3.5%) regional recurrences were found outside the ipsilateral axilla. These aberrant sites of recurrence were in concordance with the site of the harvested rSLNB in three cases. For these patients, the histologic outcome of rSLNB was possibly false negative. In four other patients, the regional recurrence was not concordant with the location of the rSLNB. One explanation could be that rSLNB in IBTR is a technically challenging procedure. As published before, injection with a larger amount of tracer leads to a higher identification rate.^32^ In this study, patients with regional recurrence had a significantly lower amount of tracer injected. Injection of a higher tracer dose might have led to identification of repeat sentinel lymph nodes in additional basins. It could also be hypothesized that lymph drainage of the IBTR might have been multidirectional, and the rSLNB identified only one basin.

In this study, 55.4% of patients with regional re-recurrence had triple-negative IBTR. Therefore, triple-negative disease seems to be a risk factor for developing regional re-recurrence. Although the numbers are small, these findings are comparable to identified risk factors for regional recurrence after primary SLNB.^33^,^34^ Patients with estrogen receptor (ER)-negative tumors (in particular, triple-negative tumors) have increased risk of developing regional recurrence after primary SLNB.^33^,^34^ Clinicians could opt for more aggressive treatment in patients with triple-negative IBTR. In this study, only 17% of patients with IBTR were treated with adjuvant chemotherapy, while the CALOR trial provided evidence of a beneficial effect of adjuvant systemic treatment on overall and disease-free survival for IBTR, especially ER-negative IBTR.^35^

Some caveats have to be considered regarding this study. Given the small number of regional events, multivariable analyses were inappropriate and limited statistical conclusions could be made. Furthermore, a randomized controlled trial comparing rSLNB with ALND would have been preferable. On the other hand, such a trial would most probably be underpowered due to the low incidence of recurrent breast cancer and regional recurrence after recurrent breast cancer. Despite these limitations, the present study is unique in the fact that follow-up data were available for a large cohort of patients with IBTR and negative rSLNB. No other studies on regional recurrence after negative rSLNB have included such a large patient population. Furthermore, this is a multicenter study, including data from different types of hospital in The Netherlands with different breast cancer volumes. Going forward, further research is encouraged in the field of rSLNB to optimize the prognostic value of this procedure, but on the basis of present evidence, cALND can be safely omitted after negative rSLNB.

## Conclusions

The 5-year risk of developing regional recurrence after negative rSLNB without subsequent ALND in patients with IBTR is less than 5%, with only 1% being located in the ipsilateral axilla. This low relapse rate provides further evidence that rSLNB is a safe primary staging method in IBTR, in terms of regional recurrence. Based on these data, we suggest to adopt rSLNB as standard of care in IBTR and to omit ipsilateral ALND.

## Electronic supplementary material

Below is the link to the electronic supplementary material.

Supplementary material 1 (DOCX 22 kb)
