# Supplementary material for: Risk of Regional Recurrence After Negative Repeat Sentinel Lymph Node Biopsy in Patients with Ipsilateral Breast Tumor Recurrence
Source: Ann Surg Oncol. 2018 Mar 1;25(5):1312–21. doi: 10.1245/s10434-018-6384-y (PMC5891565; doi:10.1245/s10434-018-6384-y)
Supplement: Supplementary file 1 — Supplementary material 1 (DOCX 22 kb) [file 10434_2018_6384_MOESM1_ESM.docx]

**List of supporting information**

**Variables used:**

Age, tumor and axillary surgery, nodal classification, tumor stage, receptor status and adjuvant therapy of primary tumor and IBTR; time interval from primary surgery to IBTR; repeat SN tracer amount.

**Detailed information primary treatment radiotherapy and location:**

Of these patients, nine were treated with adjuvant radiotherapy to the axilla and/or regional basins of which seven patients to the ipsilateral axilla and supraclavicular region, one patient to the supraclavicular region and one patient to the internal mammary chain region. None of these nine patients experienced regional recurrences after IBTR. Information of adjuvant radiotherapy to the regional basins was unknown in 59 patients, of which two experienced a regional recurrence after IBTR.
